# Editorial: Immunometabolism: bridging the gap between immunology and nutrition

**DOI:** 10.3389/fnut.2024.1436894

**Published:** 2024-06-12

**Authors:** Paulo José Basso, Thierry Gauthier, Francisca Palomares, Soledad López-Enríquez, Sue Tsai

**Affiliations:** ^1^Department of Medical Microbiology and Immunology, University of Alberta, Edmonton, AB, Canada; ^2^Mucosal Immunology Section, National Institutes of Dental and Craniofacial Research (NIDCR), National Institutes of Health, Bethesda, MD, United States; ^3^Department of Medical Biochemistry, Molecular Biology, and Immunology, School of Medicine, Universidad de Sevilla, Seville, Spain; ^4^Institute of Biomedicine of Seville (IBiS), Virgen del Rocío University Hospital, Virgen Macarena University Hospital, University of Seville, CSIC, Seville, Spain

**Keywords:** diet, inflammation, probiotics, prebiotics, polyphenols, dietary minerals, mitochondria, animal models

## 1 Introduction

Over the past 15 years, the field of immunometabolism has seen significant advancements, delving into the complex interactions between the immune system and metabolic processes to modulate immune function. This rapidly expanding field has depicted how metabolic pathways influence different immune cell subsets in both health and disease as well as how genes, transcripts, and proteins interact with these pathways.

A portfolio of evidence indicates that quiescent and semi-quiescent (e.g., memory cells) immune cells primarily rely on oxidative phosphorylation (OXPHOS) to produce relatively high amounts of ATP, to support the maintenance of cellular homeostasis during periods of low activity, and to store energy that can be mobilized for imminent activation. Upon activation, immune cells undergo a metabolic shift to aerobic glycolysis, also known as the Warburg effect (1). This shift allows for rapid but low-efficiency ATP production and provides the necessary biosynthetic precursors to support the increased demands of cell proliferation, cytokine production, and other immune functions essential for an effective response to pathogens or tissue damage. Moreover, additional elements are necessary to sustain cellular anabolism during the activation process. Notably, the reduced form of nicotinamide adenine dinucleotide phosphate (NADPH), a predominant cytoplasmic co-factor, is an key for anabolic reactions and plays a significant role in both oxidative and anti-oxidative processes. In this edition, Ting et al. elegantly outlined the significance of this co-factor in metabolic reactions within the myeloid cell compartment.

Nevertheless, describing a cell's reliance on aerobic glycolysis or OXPHOS as a “yin-yang” dynamic is overly simplistic, given the complex array of metabolic processes within immune cells: glucose derivatives can be diverted to the pentose phosphate pathway (PPP); OXPHOS can be predominantly fueled by various sources, including glucose, fatty acids (FAs), and other anaplerotic reactions such as glutaminolysis and the citrate-malate shuttle; tricarboxylic acid (TCA) cycle may undergoes “breaks” leading to the accumulation of citrate, succinate and itaconate (2); mitochondrial metabolism may influence and be influenced by other non-metabolic mitochondrial functions such as cell death, autophagy, calcium flow, endoplasmic reticulum stress, superoxide production of the respiratory chain and the resulting cascade of damage induced by reactive oxygen species (ROS), and lipid trafficking through interactions with other organelles via membrane contact sites. Thus, considerable research is still ongoing to fully comprehend how the dynamics of mitochondrial activity and the role each metabolic pathway plays in the diverse functions of immune cells. Here, Liu et al. and Thind et al. provided an in-depth analysis of the latest research on the role of glutaminolysis in CD4^+^ T cells (e.g., Th1, Th17 and regulatory T cells) and how immunometabolic pathways of neutrophils affects their multiple functions in maintaining host resilience, respectively, while Trinchese et al. explored the myriad of mitochondrial functions, highlighting their primary role as energy producers and their impact on immunometabolism across a wide range of immune cells. These studies elucidate how specific metabolic pathways and mitochondrial functions interact to influence immune responses, and they identify promising new avenues for research to deepen our understanding on these mechanisms.

**Figure 1:**
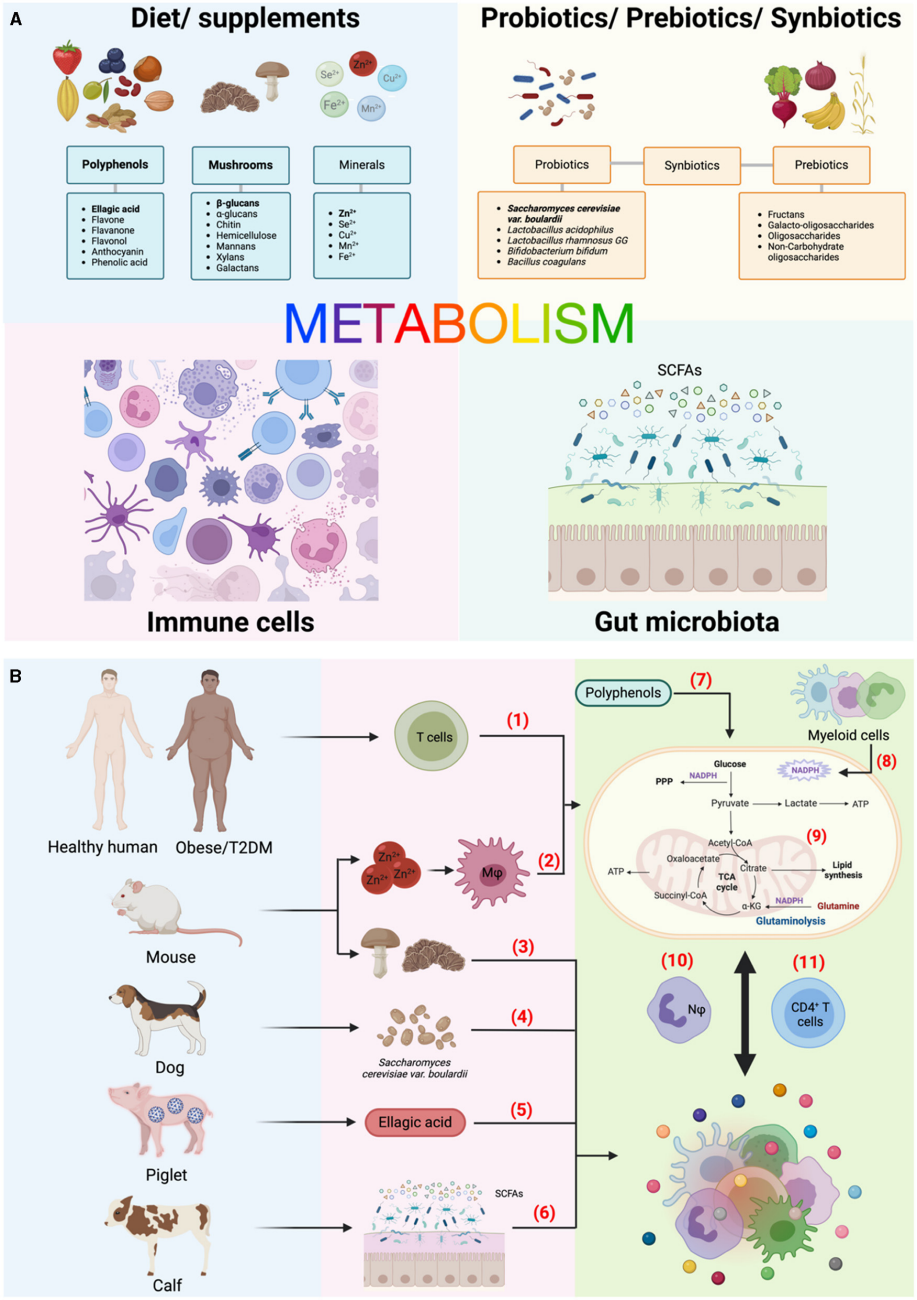
An overview of the topic landscape in this edition. **(A)** Diet, microbiota, and immunity are intricately interconnected, with metabolism serving as the pivotal link among these elements. Bold texts indicate some of the specific components investigated in this Research Topic. (**B)** Compilation of studies in this edition. The numbers highlighted in red indicate the brief scope of each study, emphasizing their experimental models and approaches to investigate their impact on immune cell metabolism and function. (1) Tibaes et al., (2) Kijima et al., (3) Case et al., (4) Garrigues et al., (5) Song et al., (6) He and Dong, (7) Ferreira et al., (8) Ting et al., (9) Trinchese et al., (10) Thind et al., (11) Liu et al. a-KG, alpha-ketoglutarate; ATP, adenine triphosphate; Cu^2+^, copper ion; Fe^2+^, iron/ferrous ion; Mφ, macrophages; Mn^2+^, manganese ion; Nφ, neutrophils; NADPH, nicotinamide adenine dinucleotide phosphate; PPP, pentose phosphate pathway; SCFAs; short-chain fatty acids; Se^2+^, selenium ion; T2DM, type 2 Diabetes mellitus; TCA, tricarboxylic acid (cycle); Zn^2+^, zinc ion or zinc (II). Created with BioRender.com.

Current investigations have aimed to understand how external biochemical, metabolic and immune factors are integrated to influence cell fate decisions. This is particularly evident with dietary factors. Different foods not only have unique compositions, but the relative amounts of certain nutrients/micronutrients can significantly alter immunometabolic signals. Indeed, Kijima et al. described that supplementation with zinc ion (Zn^2+^) can be used to treat spinal cord injury in mice, observing a dose-dependent improvement that affected both macrophages function and neuronal regeneration. Moreover, Case et al. found that pre-treatment with either mushrooms or isolated b-glucans on both murine and human macrophages improved their response to stimulation as well as increased myeloid progenitor cells in the bone marrow of mice, a process called trained immunity. Ferreira et al. and Song et al. used complementary approaches. While the former conducted a comprehensive review of polyphenols - a naturally occurring compound found primarily in plants - and their immunometabolic impact across multiple immune cell subsets, the latter used a specific polyphenol, ellagic acid, to ameliorate intestinal inflammation in piglets infected with porcine epidemic diarrhea virus (PEDV). Common sources of polyphenols include berries, nuts, olives, cocoa, olives, and certain seeds (Figure 1A). Moving in a clinical direction, Tibaes et al. outlined their protocol for the “Nutrition and Immunity (nutrIMM) study” to assess immune changes in healthy vs obese and/or individuals with type 2 diabetes upon a 4-week diet intervention.

Before reaching the bloodstream, nutrients must pass through the gut and interact with the local microbiota, undergoing various pre-processing steps. This process not only influences nutrient absorption but also impacts local and systemic immunity depending on the interspecies balance of the microbial community. Local microbiota produces several metabolites, among them the most well characterized are the short chain fatty acids (SCFAs) such as acetate, propionate and butyrate that are derived from fermentation of dietary fiber. While the interface between SCFAs and immune function has been extensively investigated (3), He and Dong provided an overview of the role of SCFAs in calves, which may have important application for “reverse translational research” in veterinary care due to the complexity of the four-compartment stomach in ruminants. Conversely, dysbiosis, an imbalance in gut microbiota, harms gut health by impairing nutrient absorption and weakening the gut barrier, leading to increased intestinal permeability and inflammation. To counteract dysbiosis, various clinical approaches, such as the use of probiotics, prebiotics, and/or synbiotics, have been employed (4). Garrigues et al. investigated the outcomes of administering *Saccharomyces cerevisae* var. *boullardii* in female dogs, observing positive changes in both microbiota composition and colostrum quality, which in turn, resulted in healthier puppies, with increased circulating levels of anti-inflammatory cytokines independently on the vaccination status.

Last, the studies presented in this Research Topic offer valuable insights into the importance of using different animal models to achieve specific research goals (Figure 1B). The selection of animal models is crucial as it impacts study outcomes due to species-specific genetic and physiological differences, relevance to human diseases, variations in immune responses, and ethical and practical considerations (5). This issue featured original research and reviews involving calves, dogs, and piglets, as well as the commonly used mouse, human, and *in vitro* approaches. This diversity suggests an unprecedented advance in the research community, despite the substantial challenges presented by each animal model.

## 2 Conclusion and perspectives

The emerging field of immunometabolism reveals how dietary and metabolic interventions can enhance immune health, offering new avenues for therapeutic development. The covered literature in this special edition emphasizes the critical role of nutrients in gut-immune cell axis and highlights the newfound understanding of cellular metabolic adaptability in face of different signal inputs. It particularly underscores the importance of this adaptability in immune cells, which must act swiftly against pathogens and tissue damage. Understanding these complex interactions is crucial for advancing treatment and preventive personalized medicine.

## Author contributions

PB: Writing – original draft, Writing – review & editing. TG: Writing – review & editing. FP: Writing – review & editing. SL-E: Writing – review & editing. ST: Writing – review & editing.
